# Glucose-6-phosphate dehydrogenase deficiency (G6PD) as a risk factor of male neonatal sepsis

**Published:** 2016

**Authors:** Z Rostami-Far, K Ghadiri, M Rostami-Far, F Shaveisi-Zadeh, A Amiri, B Rahimian Zarif

**Affiliations:** * Department of Biology, Sanandaj Branch, Islamic Azad University, Sanandaj, Iran; ** Nosocomial Infection Research Center, Kermanshah University of Medical Sciences, Kermanshah, Iran; *** Department of Environmental Health Engineering, School of Public Health, Kermanshah University of Medical Sciences, Iran; **** Molecular Pathology Research Center, Imam Reza Hospital, Kermanshah, University of Medical Sciences, Kermanshah, Iran

**Keywords:** sepsis, glucose-6-phosphate dehydrogenase, risk factor

## Abstract

**Introduction.**Neonatal sepsis is a disease process, which represents the systemic response of bacteria entering the bloodstream during the first 28 days of life. The prevalence of sepsis is higher in male infants than in females, but the exact cause is unknown. Glucose-6-phosphate dehydrogenase (G6PD) is an enzyme in the pentose phosphate pathway, which leads to the production of NADPH. NADPH is required for the respiratory burst reaction in white blood cells (WBCs) to destroy microorganisms. The purpose of this study was to evaluate the prevalence of G6PD deficiency in neonates with sepsis.

**Materials and methods.**This study was performed on 76 neonates with sepsis and 1214 normal neonates from February 2012 to November 2014 in the west of Iran. The G6PD deficiency status was determined by fluorescent spot test. WBCs number and neutrophils percentages were measured and compared in patients with and without G6PD deficiency.

**Results.**The prevalence of the G6PD deficiency in neonates with sepsis was significantly higher compared to the control group (p=0.03). WBCs number and neutrophils percentages in G6PD deficient patients compared with patients without G6PD deficiency were decreased, but were not statistically significant (p=0.77 and p=0.86 respectively).

**Conclusions.**G6PD deficiency is a risk factor of neonatal sepsis and also a justification for more male involvement in this disease. Therefore, newborn screening for this disorder is recommended.

## Introduction

Neonatal sepsis is a disease process, which represents the systemic response of bacteria entering the bloodstream during the first 28 days of life, which was confirmed by positive blood cultures in the presence of clinical and laboratory findings. It is a very serious condition and if not diagnosed and treated quickly, can lead to shock, multiple organ dysfunction, permanent disability or death [**1**,**2**].

According to the onset of age, neonatal sepsis is divided into early-onset sepsis (≤ 3 days of birth) and late-onset sepsis (after 3 days). The early-onset sepsis usually results from organisms acquired intrapartum but late-onset sepsis is usually acquired from the environment [**1**,**3**]. Neonatal sepsis occurs in one to four cases per 1000 live births in the developed countries, while it has been 10 times greater in many developing countries [**4**]. Group B Streptococcus, Escherichia coli, coagulase-negative Staphylococcus, Staphylococcus aureus, Klebsiella, Pseudomonas and Enterobacter are the most common cause of neonatal sepsis [**5**]. The medical advances in the treatment of sepsis reduce the incidence of mortality from 90% to 15-30%. Diagnosis of neonatal sepsis remains one of the challenges that pediatrics are facing, because clinical findings are nonspecific and vague [**4**].

The prevalence of sepsis is higher in male neonates than in females but the exact mechanism is unclear, although some factors such as x-linked genes of immune system and hormonal differences were cited [**6**]. While a positive blood culture is still a gold standard of sepsis diagnosis, the preparation of results take time and there are possibilities of contamination, false-positive and false-negative results [**7**]. Glucose-6-phosphate dehydrogenase (G6PD) is one of the enzymes of many organisms including humans, which is expressed in all tissues and plays a key role in the process of glucose metabolism [**8**]. G6PD catalyzes the first reaction of the pentose phosphate pathway that converts glucose to pentose carbohydrates. In addition to this reaction, nicotinamide adenine dinucleotide phosphate (NADPH) is produced [**4**]. One function of NADPH is the reduction of glutathione. Reduced glutathione plays a major role in the detoxification and neutralization of oxidants [**9**].

Phagocytosis and then respiratory burst reactions are the main mechanism of destroying invasive microorganisms by neutrophils and other white blood cells (WBCs). In the beginning of the phagocyte antibacterial activity, reactive oxygen species (ROS) produced by NADPH-oxidase enzyme use oxygen and NADPH. ROS are major factors of respiratory burst reactions, because their strong activities can destroy microorganisms [**10**]. Although required NADPH for ROS production can be produced from other pathways, a hypothesis proposed that G6PD deficiency reduces NADPH content of WBCs, and through this way reduces the production of ROS and the ability to fight against invasive bacteria [**11**].

Little research has shown the relationship between G6PD deficiency and increased incidence of certain infections such as viral hepatitis and typhoid fever [**12**,**13**]. Also some studies investigated the importance of G6PD deficiency in sepsis, but more focus was on hemolysis aspects of erythrocytes as a result of medical treatments against microorganisms in patients [**11**]. 

## Material and methods

### Study design

This cohort study was conducted between 12th February 2011 and 27th November 2014 on 76 neonates with sepsis and 1124 normal neonates without any medical problems, as the control group. Only neonates with suspected sepsis and age less than 28 days were included [**5**]. Our study complies with the Declaration of Helsinki. 

The inclusion criteria consisted of clinical signs such as reduced activity, lethargy, hypotonia, hyporeflexia, apnea, cyanosis, respiratory distress, irritability, hypothermia, hyperthermia, diarrhea, vomiting, abdominal distension and poor breastfeeding and laboratory findings such as positive blood culture results, thrombocytopenia, increase or decrease of WBCs, increase of C-reactive protein (CRP) and erythrocyte sedimentation rate (ESR) and so on [**14**]. Blood culture is a gold standard for the diagnosis of sepsis, but since the positive result of blood culture may be false due to contamination, according to pediatric examination, only neonates have clinical and laboratory symptoms and their blood culture results were positive and considered as a real sepsis [**8**]. 1124 healthy neonates without any medical problems were considered as the control group.

### Bacterial identification

1 ml blood samples were drawn aseptically from all neonates with suspected sepsis, inoculated into a heart infusion broth, incubated at 37°C, and was daily checked for signs of bacterial growth up to 7 days. For a positive broth culture, sub-cultures were done on Mac Conkey agar, blood agar and chocolate agar then incubated at 37°C for 24 h. Blood culture broths with no bacterial growth after 7 days were sub-cultured and if no growth occurred, were reported as a negative. Bacterial isolates were identified by colony morphology, gram staining and standard biochemical tests.

### Fluorescent spot test

1 ml blood samples were taken from all the patients and controls and collected in tubes containing EDTA. A fluorescent spot test method and a commercial kit were used to determine the G6PD deficiency status. G6PD enzyme activity for the conversion of the glucose-6-phosphate to 6-phosphogluconate and the reduction of NADH to NADPH2 is the basis of this qualitative method. NADPH2 have fluorescence under UV light (365 nm). The presence and absence of fluorescence was considered an active and deficient G6PD enzyme respectively. 

### Data analysis

SPSS version 16 software was used for the statistical analysis. The Chi square test was used to determine the relationship between dependent and independent variable. P<0.05 was considered statistically significant.

## Results

This survey was performed in the neonatal intensive care unit (NICU) of Imam Reza University Hospital of Kermanshah in the west of Iran. From 76 newborns with sepsis, 41 were males (53.9%) and 35 were females (46.1%); also, 43 had an early-onset sepsis (56.6%) and 33 had a late-onset sepsis (43.4%). Nine species of bacteria were isolated during the study, the number and frequency of each species being shown in **Table 1**. The frequency of isolated gram-negative compared to the gram-positive bacteria was higher (**Table 2**). The most frequent isolated bacteria in both groups of patients with and without G6PD deficiency was Staphylococcus aureus. Blood cultures, in which coagulase-negative Staphylococci were not isolated for more than once or separated from the mixture of bacteria, were considered a contamination and excluded from the results. There was a statistically significant correlation between the prevalence of G6PD deficiency and sepsis in the neonates (p=0.03) (**Table 3**) in this study. 

As **Table 3** shows, the prevalence of G6PD deficiency compared in the two groups of female neonate patients and female neonate controls, were not statistically significant (p=0.39), but were statistically significant (p=0.03) in male neonates. The mean WBCs in the G6PD-deficient patients and the G6PD normal patients were 11.6 and 10.7 cells/ mcL respectively (p= 0.77); also, the mean neutrophil percentages in G6PD-deficient patients and G6PD normal patients were 54.86% and 53% respectively (p=0.86). 

**Table 1:**
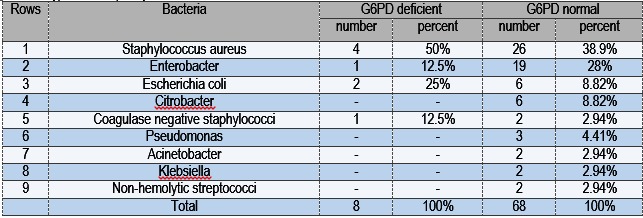
Types and frequency of bacteria isolated from blood cultures

**Table 2:**
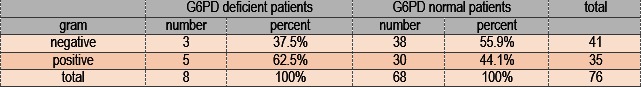
Distribution of bacteria isolated from blood cultures, according to the Gram staining

**Table 3:**
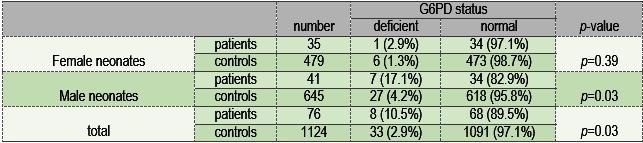
Prevalence of G6PD deficiency in patients and controls according to gender

## Discussion

The G6PD is a key enzyme in the glucose metabolism. G6PD converts glucose-6-phosphate to 6-phosphogluconolactone combined with the reduction of NADP to NADPH. Glutathione reduced by NADPH is essential for neutralizing the oxidant components [**15**]. G6PD deficiency is the most common human enzyme defect and 400 million people in the world are affected [**9**]. G6PD deficiency may clinically manifest as a neonatal jaundice and acute hemolytic anemia following ingestion of fava beans (favism) and certain drugs [**16**,**17**].

So far, Many studies have been performed so far on the relationship between the G6PD deficiency and the susceptibility to development of sepsis in neonates [**11**,**18**]. In our study, the prevalence of G6PD deficiency in male neonates in both groups of patients and controls was significantly more than in the female neonates of same group. This is explained by the x-linked recessive inheritance of G6PD deficiency, because this pattern of inheritance mostly affects males [**19**]. Also, the prevalence of G6PD deficiency was significantly higher in patients compared to controls. There are reports of an increased prevalence of G6PD deficiency in association with some of the infections [**13**].

As previously mentioned, neonatal sepsis occurs more in males compared to females. The exact mechanism of higher susceptibility of sepsis in male neonates is not entirely clear but some reasons, such as the x-linked genes involved in the immune system and hormonal differences were mentioned [**6**]. We hypothesized that one of the reasons for the higher prevalence of sepsis among male neonates may be due to G6PD deficiency. Since the G6PD deficiency is the most common genetic defect in the world and its mode of inheritance is x-linked recessive, many male newborns were affected [**20**]. On the other hand, as this study showed, the frequency of G6PD deficiency in neonates with sepsis was higher than in controls. Therefore, we concluded that the G6PD deficiency is one of the reasons for the higher neonates’ susceptibility of developing sepsis, so, G6PD deficiency can be considered a risk factor of male neonatal sepsis.

The exact mechanisms of increased susceptibility of sepsis in G6PD deficient neonates are not clear and require further investigations, but one hypothesis is that reducing the synthesis of ROS resulting from the lack of NADPH due to G6PD deficiency may be one of these mechanisms. Laboratory studies showed a G6PD deficiency decrease production of NADPH in neutrophils [**21**]. Cooper and colleagues reported that the G6PD deficiency is associated with the decrease in the production of ROS in WBCs of patients, which increases the overall chance of the infection [**22**]. The other possible mechanisms that might explain the increased prevalence of sepsis among G6PD-deficient neonates was increased serum iron concentration due to lysis of erythrocytes. Some studies showed a relationship between the elevation of blood iron (hyperferremia) and serious infections. For example, the increased prevalence of sepsis in the meningitis patient as a result of intramuscular injection of iron was reported [**23**].

In contrast, other studies showed that the incidence of sepsis in premature infants with a gestational age of 27-32 weeks was higher than term infants and it was not related to the G6PD deficiency in patients [**18**]. Ardati and colleagues concluded in their research that the reduced activity of G6PD to as low as 23% of normal, does not affect the neutrophil function [**24**]. Also Zareifar and colleagues investigated the prevalence of G6PD deficiency in neonates with sepsis and reported that the G6PD deficiency was not associated with the risk of sepsis infection [**18**].

The reduction in the number of leukocytes (leukopenia) and neutrophils (neutropenia) is one of the non-specific symptoms of sepsis. In general, the number of leukocytes increased during sepsis (leukocytosis), but in some cases, the number of leukocytes is reduced [**25**]. This study showed that the number of the WBCs in the G6PD deficient patients was lower compared to the patients without enzyme deficiency; however, this decrease was not significant. Hsieh and colleagues demonstrated that the G6PD deficient epithelial cells infected with Staphylococcus aureus compared to normal infected epithelial cells have an accumulation of more oxidants and a higher apoptosis rate during infection [**21**]. This may be due to the decrease in NADPH production as a result of G6PD deficiency, which prevents the effective neutralization of oxidants. It can be assumed that during the sepsis, neutrophils phagocytosis of bacteria and respiratory burst reactions occur. However, because of G6PD deficiency, not enough NADPH is produced to neutralize all the oxidants produced. Therefore, these oxidants cause damage and apoptosis of neutrophils. However, due to the small sample size and the lack of measurement of NADPH, this conclusion is not conclusive and the repeating of this study with a larger population and measurement of NADPH levels is recommended.

## Conclusion

G6PD deficiency can be considered a risk factor of neonatal sepsis especially in male neonates. Therefore, it is suggested that in areas in which the prevalence of sepsis among neonates is high, and in areas in which the prevalence is lower, only male neonates are screened for G6PD deficiency.

### Acknowledgements

The authors sincerely thank all the health centers and staff for their assistance.

### Disclosure

The authors have no financial relationships relative to this article to disclose.
